# Supplementary material for: Necroptosis in both tumour and stromal compartments determines responsiveness to immunogenic cell death-based immunotherapy
Source: Nat Commun. 2026 Mar 6;17:3597. doi: 10.1038/s41467-026-70133-8 (PMC13096328; doi:10.1038/s41467-026-70133-8)
Supplement: Supplementary file 1 — Supplementary Information [file 41467_2026_70133_MOESM1_ESM.pdf]

SUPPLEMENTARY FIGURES

Supplementary Fig. 1

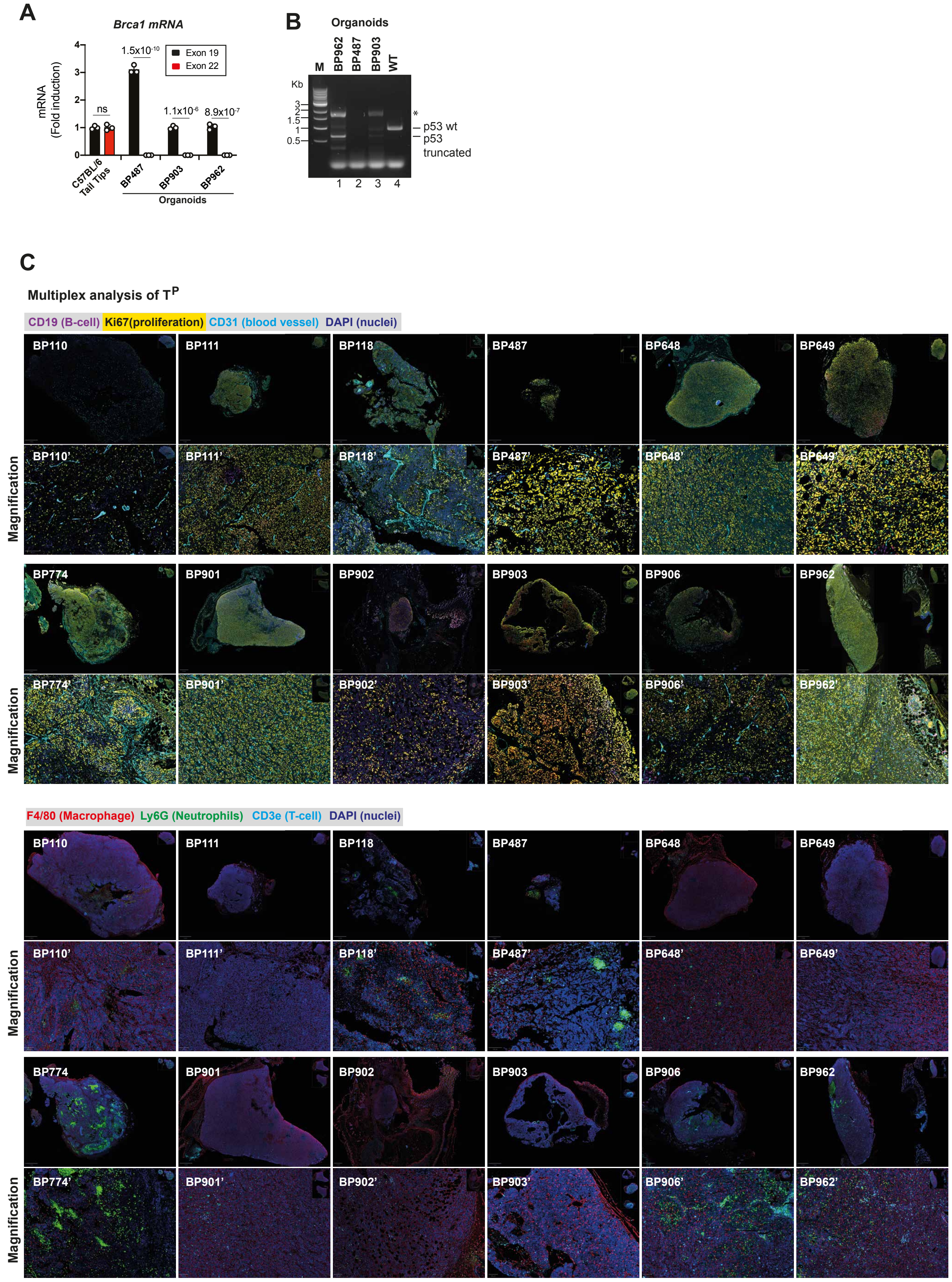

Supplementary Fig. 1. Development of a transplantation-based mouse model of TNBC.

(A) Validation of the BRCA1 and p53 status. Relative mRNA expression of the floxed *Brca1* region in tail tips from wildtype C57BL/6J mice and tumour-derived organoids. Each column represents three independent samples (n = 3). Graphs show mean ± SD. *p* values were calculated using two-way ANOVA (Sidak's multiple comparison test).

(B) PCR analysis evaluating the p53 status of the indicated tumour-derived organoids and control wild-type organoids. Image is representative of two independent biological replicates.

(C) Multiplex analysis highlighting that the individual tumours greatly differ in their composition. Representative images from individual primary BP tumours showing B cells (CD19), blood vessels (CD31), Neutrophils (Ly6G), T cells (CD3e) and Macrophages (F4/80). Each image represents one primary tumour (one animal). Scale bar 1 mm and 100 µm.

Source data are provided as a Source Data file and in

Zenodo: https://doi.org/10.5281/zenodo.18130193

Supplementary Fig. 2

A

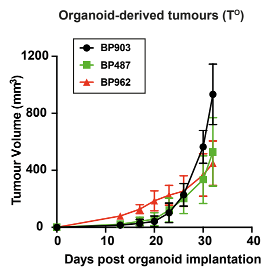

B

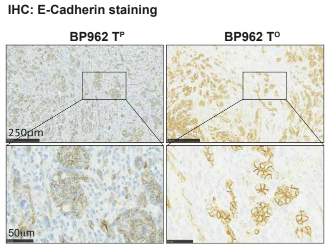

C

Histological characteristics on haematoxylin and eosin staining								
Organoid Model	Tumour Type	Nuclear Pleomorphism	Tubule Formation	Mitotic index	Grade	Cellular morphology	Necrosis	Stroma
BP903	T^P	P3	T3	M3	3	Ovoid predominant	<1%	Densely arranged CAFs with plump nuclei
BP487	T^P	P3	T2	M2	2	Pure ovoid	2%	CAF-rich
BP962	T^P	P3	T3	M3	3	Mix of spindle and oval cells	10%	Small number of plump CAFs

D

Tumour line	AIMS Subtype
BP903 T^P	Basal
BP903 T^O	
BP487 T^P	Basal
BP487 T^O	
BP962 T^P	Basal
BP962 T^O	
Mammary gland	Normal

E

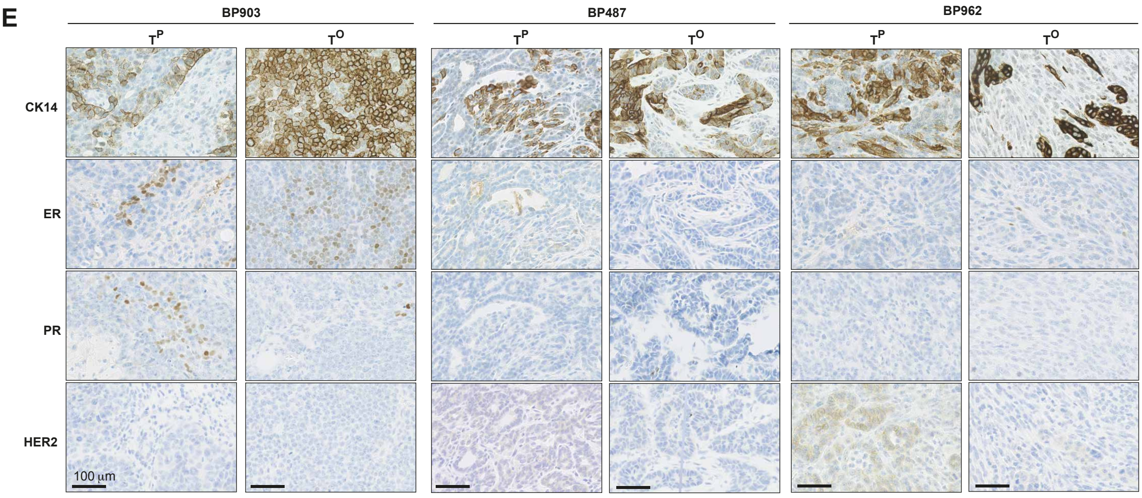

F

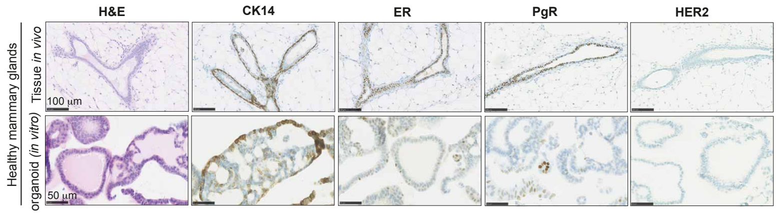

G

Hormone Receptor Classification		IHC Marker Score							
		ER			PgR				
Organoid Model	Tumour Type	Intensity score	Proportion score	Total score	Intensity score	Proportion score	Total score	Her2	CK14
BP903	T^P	2	2	4/8 Positive	2	3	5/8 Positive	0 Negative	50% Positive
	T^O	1	4	5/8 Positive	3	1	4/8 Negative	0 Negative	70% Positive
BP487	T^P	1	1	2/8 Negative	0	0	0/8 Negative	0 Negative	20% Positive
	T^O	0	0	0/8 Negative	2	1	3/8 Negative	0 Negative	10% Positive
BP962	T^P	1	1	2/8 Negative	0	0	0/8 Negative	1+ Negative	20% Positive
	T^O	1	1	2/8 Negative	0	0	0/8 Negative	0 Negative	20% Positive

H

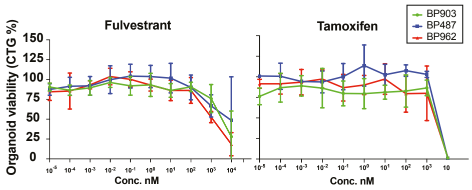

Supplementary Fig. 2. Organoid-derived tumours faithfully recapitulate their corresponding primary tumour.

(A) Organoids derived from primary tumours retain their tumourigenic potential upon engraftment into naïve C57BL/6J female recipients – BP903 (n=5), BP487 (n=5), BP962 (n=9). Graphs show mean ± SD.

(B) Representative IHC images showing E-cadherin staining (brown) in BP962 T^Ps and their corresponding T^Os. The T^P image represents a single primary tumour (one animal), whereas T^O images are representative of three independent tumours. Scale bar 250 µm (top panel) and 50 µm (bottom panel).

(C) Table summarising the histological characteristics of the H&E sections of the indicated T^Ps.

(D) Table showing AIMS classification of T^Ps and their corresponding T^Os.

(E) Representative IHC images showing the expression of basal-like markers cytokeratin 14 (CK14), estrogen receptor (ER), progesterone receptor (PgR), and human epidermal growth factor receptor 2 (HER2) in the indicated T^Ps and T^Os. The T^P image represents a single primary tumour (one animal), whereas T^O images are representative of three independent tumours. Scale bar: 100 µm.

(F) Representative H&E and IHC staining of CK14, ER, PgR and HER2 in the indicated organoids. Positive control staining is shown from a single mammary gland (one animal) and one established primary mammary gland organoid line. Scale bar 100 µm (top panel) and 50 µm (bottom panel).

(G) Table indicating the scores for ER, PgR and HER2, as well as CK14 expression in the indicated T^Ps vs T^Os. The T^P score represents a single primary tumour (one animal), whereas T^O score is representative of three independent tumours.

(H) Cell viability assay of organoids treated with the indicated agents. Cell viability was measured using Cell Titre Glo (CTG). Graphs show mean ± SD, n=2 independent biological repeats.

Source data are provided as a Source Data file and in

Zenodo: https://doi.org/10.5281/zenodo.18130193

Supplementary Fig. 3

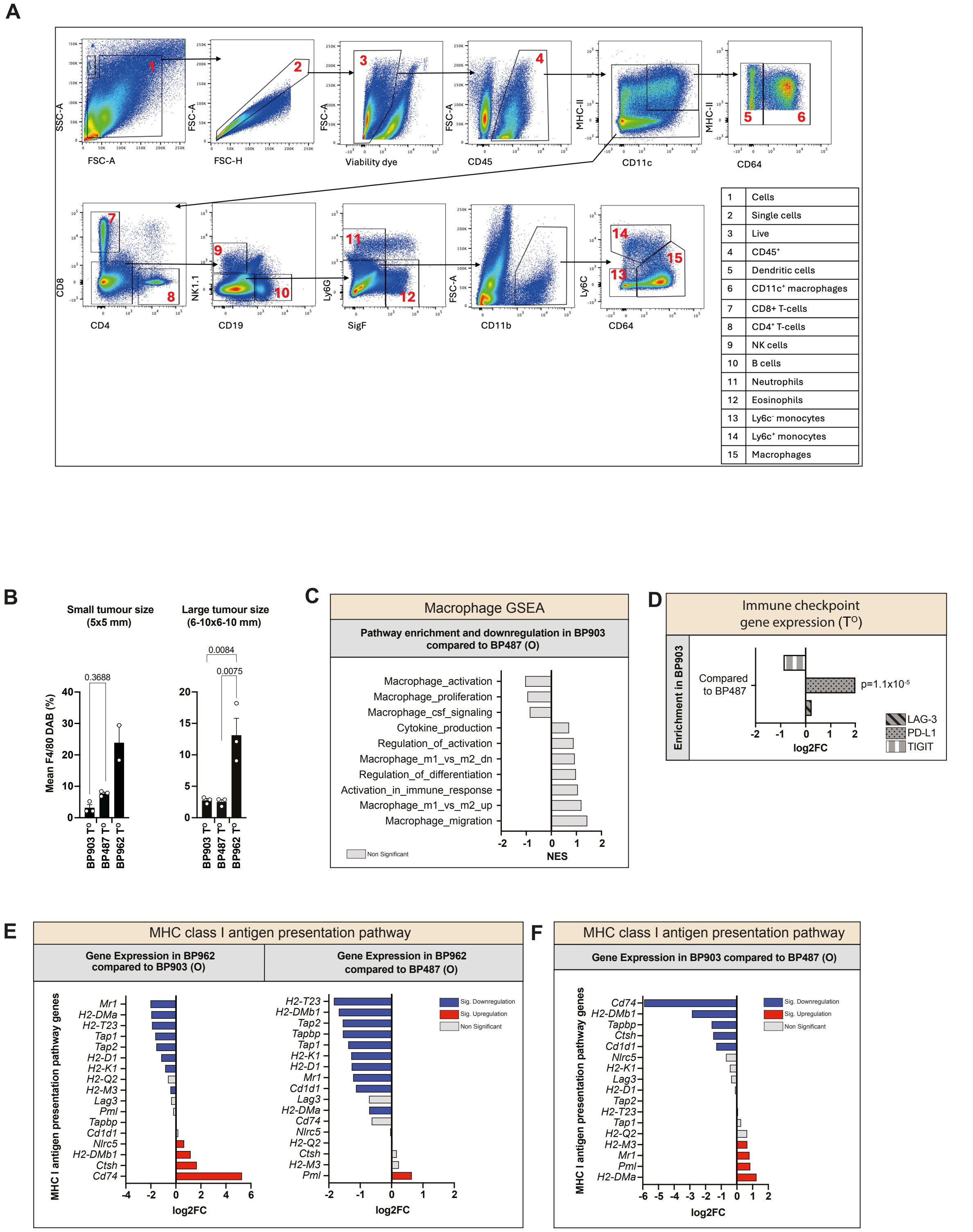

Supplementary Fig. 3. T^{O}s exhibit diverse immune landscape profiles

(A) FACS gating strategy, related to Fig 3. 1) Cells, 2) Single cells, 3) Live cells, 4) CD45^{+} cells, 5) Dendritic cells, 6) CD11c^{+} macrophages, 7) CD8^{+} T-cells, 8) CD4^{+} T-cells, 9) NK cells, 10) B cells, 11) Neutrophils, 12) Eosinophils, 13) Ly6c^{-} monocytes, 14) Ly6c^{+} monocytes, 15) Macrophages.

(B) Quantification of F4/80+ cells in the indicated T[O]s. Each dot represents one independent tumour (one animal).

(C) Gene set enrichment analysis (GSEA) of the indicated macrophage gene sets in the indicated T[O]s. Grey bars represent non-significant results based on an adjusted *p*-value ($p < 0.05$). See Supplementary Data 2 for further information.

(D) Differential analysis of the indicated immune checkpoint gene expression in BP903 T[O]s versus BP487 T[O]s.

(E,F) Differential analysis of MHC class I antigen presentation pathway genes. Red bars represent statistically upregulated gene sets, blue bars represent statistically downregulated gene sets and grey bars represent non-significant gene sets.

Data show mean ± SEM, *p* values were calculated using one-way ANOVA (Tukey's multiple comparisons test) (B). Statistical testing for RNA-Seq data (D) was performed using quasi-likelihood negative binomial generalized log-linear model (function glmQLFTest from edgeR).

Source data are provided as a Source Data file and in

Zenodo: https://doi.org/10.5281/zenodo.18130193

Supplementary Fig. 4

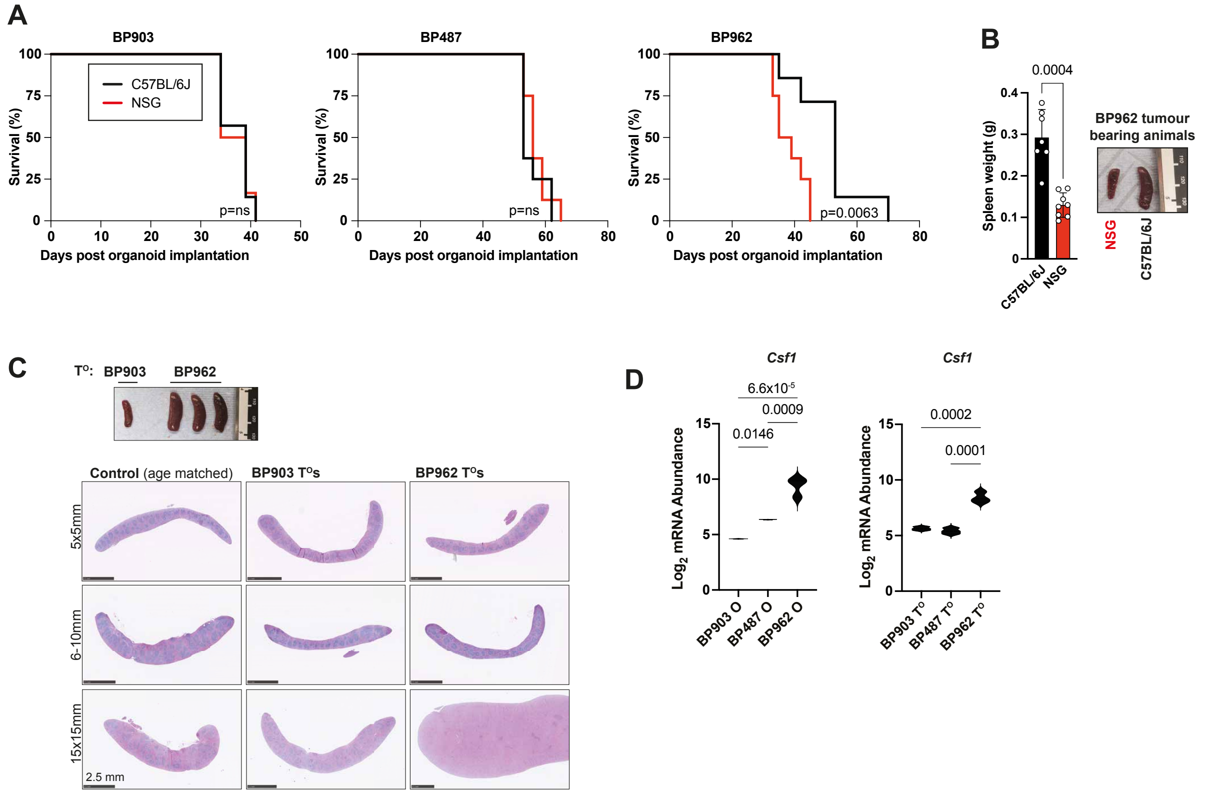

Supplementary Fig. 4. Differential growth of T^{O}s in immunocompromised mice

(A) Survival curves of BP487, BP903 and BP962 tumour-bearing NSG and C57BL/6J mice. BP903: NSG (n=6), C57BL/6J (n=7), BP487: NSG (n=8), C57BL/6J (n=8), BP962: NSG (n=8), C57BL/6J (n=7).

(B) Graph showing spleen weights of BP962 tumour-bearing NSG and C57BL/6J mice, respectively. Right: representative image of spleens from corresponding groups.

(C) Representative images and H&E stains of spleens from age-matched, tumour-free C57BL/6J mice (n=3) and mice bearing BP903 (n=3) and BP962 (n=3) tumours of varying sizes.

(D) Violin plot of mRNA expression of *Csf1* in the indicated organoids and T^{O}s (n=3 independent samples per organoid or three independent tumours per T^{O}s).

Data show mean ± SD. *p* values were calculated using Log-rank (Mantel-Cox test) (A), Welch's t test, two-tailed (B) and one-way ANOVA (Bonferroni multiple comparison test) (D).

Source data are provided as a Source Data file and in

Zenodo: https://doi.org/10.5281/zenodo.18130193

Supplementary Fig. 5

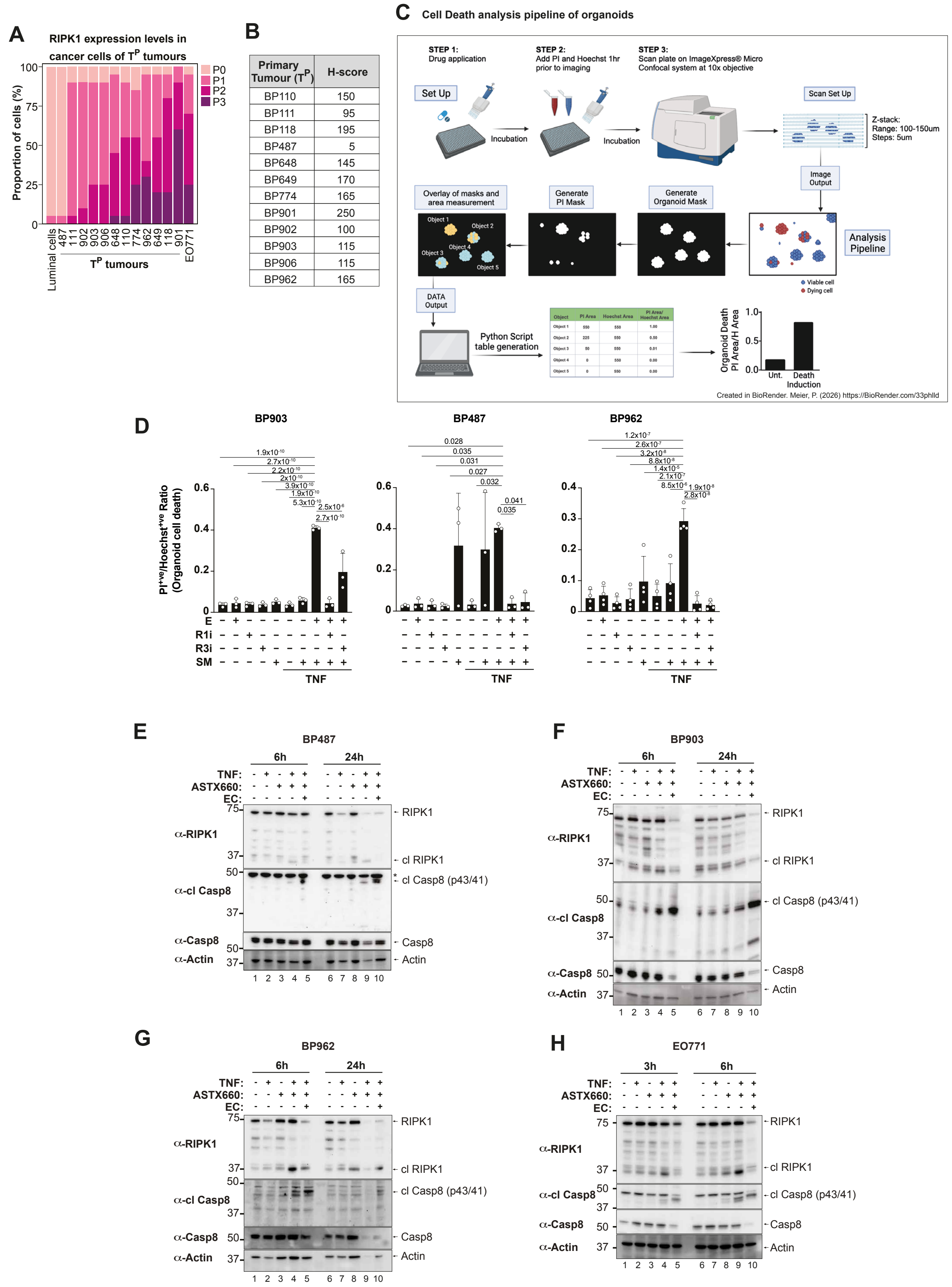

Supplementary Fig. 5. RIPK1-mediated cell death in BP903, BP487 and BP962 organoids

(A) Stacked histogram of RIPK1 protein expression in cancer cells of individual T^Ps.

(B) Table listing T^Ps, ranked in order of their H-scores.

(C) Schematic representation of the ImageXpress pipeline used to quantify cell death in organoids. Figure created with BioRender.com under the ICR – Division of Breast Cancer Research institutional license. Created in BioRender. Meier, P. (2026) https://BioRender.com/33phlld.

(D) Cell death assays of organoids treated for 8h with the indicated agents. Each dot represents one independent biological replicate. Data show mean ± SD. *p* values were calculated using one-way ANOVA with Tukey's multiple comparisons test.

(E-H) Western blot analysis of treated organoids and EO771 cells. Western blot analysis of lysates from the indicated cells, treated with the respective agents. Samples derive from the same experiment. RIPK1 and cleaved caspase-8 were detected on separate membranes processed in parallel. In E-G panels, RIPK1 was re-probed for caspase-8, and cleaved caspase-8 was re-probed for Actin (loading control). In (H), RIPK1 was stripped and re-probed for Actin, and cleaved caspase-8 for caspase-8. Representative data of at least 2 independent biological experiments is shown.

Source data are provided as a Source Data file and in

Zenodo: https://doi.org/10.5281/zenodo.18130193

Supplementary Fig. 6

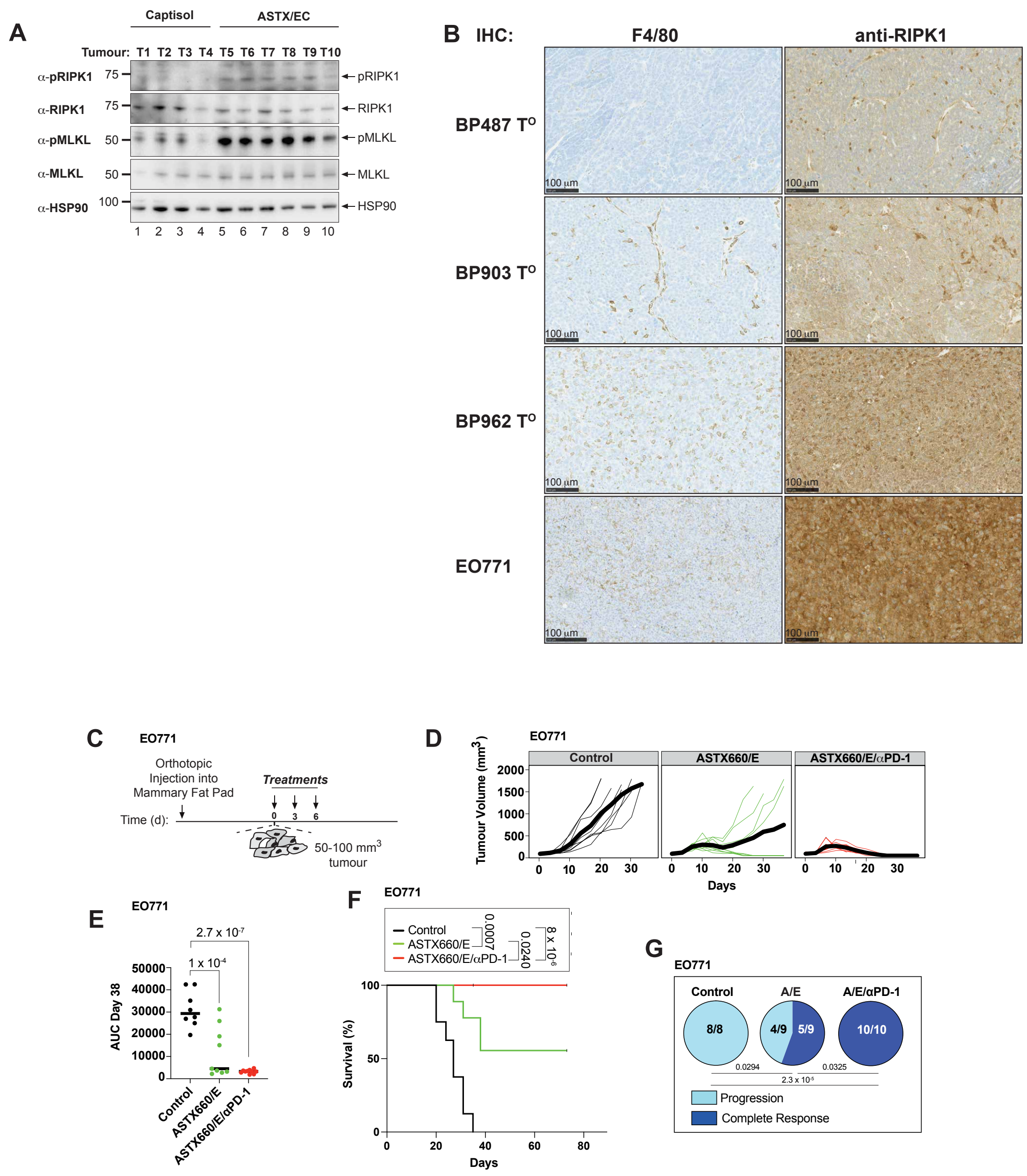

Supplementary Fig. 6. Necroptosis in tumours and other tumours that express high levels of RIPK1.

(A) ASTX660/E induces necroptosis in the tumour. Western blot analysis of lysates from BP962 tumour bearing-C57BL/6J mice, treated with captisol or ASTX660/E. Tumours were harvested 24 hrs after treatment. Each lane represents an individual tumour (T1–T10) from the same experiment. Samples were run on separate membranes processed in parallel. The P-RIPK1 membrane was stripped and re-probed for RIPK1. HSP90 served as the loading control.

(B) Representative IHC images of F4/80+ and RIPK1 expression of the indicated tumours. T°s were derived from more than three independent animals. Scale bar = 100 µm.

(C) Schematic representation depicting the treatment regimen of E0771 tumour bearing-C57BL/6J mice.

(D) Tumour growth curves of E0771 derived tumours: Control (n = 8), A/E (n=9) and A/E/αPD-1 (n=10). Each line represents one animal and thick lines represent average tumour growth.

(E) Tumour growth kinetics (days 0-38) of mice treated as in (C), measured by the area under the curve (AUC). Each point represents the AUC of individual mice from (D).

(F) Kaplan-Meier survival curves of E0771 tumour-bearing animals, treated as in (C).

(G) Pie charts depicting the proportion of mice from (F), which progressed, or fully responded to the indicated treatments.

A=ASTX660; E=emricasan

p values were calculated using ordinary one-way ANOVA with Tukey's multiple comparisons (E), Log-rank (Mantel-Cox) test (F) and Fisher's exact test, two-sided (G).

Source data are provided as a Source Data file and in

Zenodo: https://doi.org/10.5281/zenodo.18130193

Supplementary Fig. 7

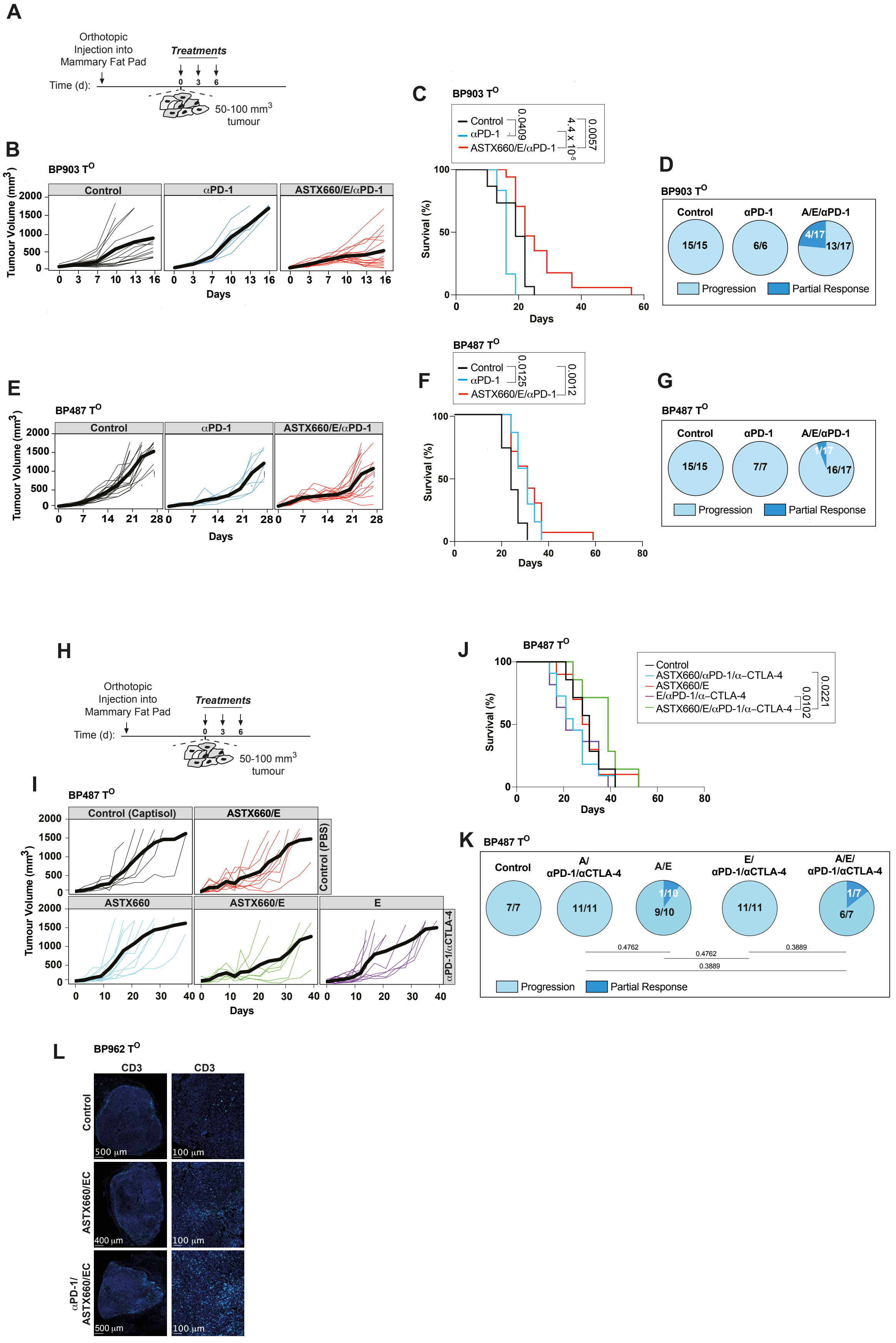

Supplementary Fig. 7. Cold tumours do not profit from immunotherapy in combination with IAP antagonists.

(A) Schematic representation depicting the treatment regimen of BP903 and BP487 tumour bearing females.

(B) Tumour growth curves for BP903 T^Os treated as depicted in (A): Control (n=15), αPD-1 (n=6), and ASTX660/E/αPD-1 (n=17). Each line represents one animal and thick lines represent average tumour growth.

(C) Kaplan-Meier survival curves of BP903 tumour-bearing females, treated as in (A).

(D) Pie charts showing the proportion of tumour-bearing mice that experienced progression, or partial response to the indicated treatment from (A).

(E) Tumour growth curves of BP487 T^Os, treated as in (A): Control (n=15), αPD-1 (n=7), and ASTX660/E/αPD-1 (n=17). Each line represents one animal and thick lines represent average tumour growth.

(F) Kaplan-Meier survival curves of BP487 tumour-bearing mice, treated as in (A).

(G) Pie charts showing the proportion of tumour-bearing mice that experienced progression, or partial response to the indicated treatment from (A).

(H) Schematic depicting the treatment regimen of BP487 tumour bearing C57BL/6J mice.

(I) Tumour growth curves for BP487 T^Os, treated as in (H): Control (n=7), ASTX660/αPD-1/αCTLA-4 (n=11), ASTX660/E (n=10), E/αPD-1/αCTLA-4 (n=11) and ASTX660/E/αPD-1/αCTLA-4 (n=7). Thick lines represent average tumour growth.

(J) Kaplan-Meier survival curves of BP487 tumour-bearing animals, treated as in (H).

(K) Pie charts showing the proportion of tumour-bearing mice that experienced progression, partial response, or full response to the indicated treatments.

(L) Multiplex analysis. Representative images from BP962 tumours treated as shown in Fig. 5L, depicting T cells (CD3+) following treatment. BP962 T^Os were derived from three independent animals. Scale bar 400/500 µm and 100 µm.

p values were calculated using Log-rank (Mantel-Cox test) (C, F, J), Fisher's exact test, two-sided (K).

A=ASTX660; E=emricasan

Source data are provided as a Source Data file and in

Zenodo: https://doi.org/10.5281/zenodo.18130193

Supplementary Fig. 8

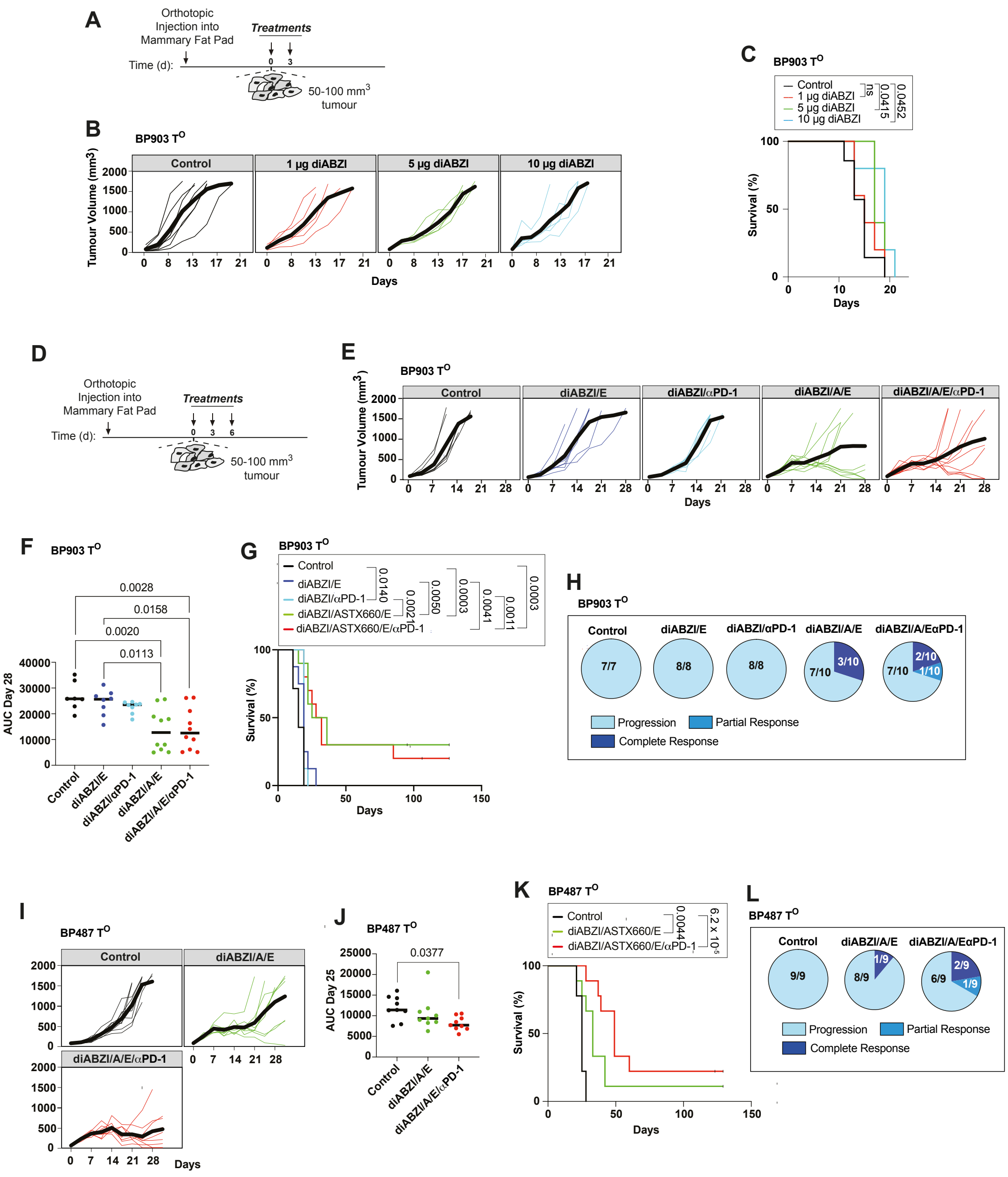

Supplementary Fig. 8. Boosting the immunogenicity of IAP antagonism through 'viral mimicry'

(A) Schematic representation depicting the treatment regimen of BP903 tumour-bearing mice.

(B) Tumour growth curves of BP903 T^{O}s, treated with various concentrations of diABZI. Each line represents one animal and thick lines represent average tumour growth.

(C) Kaplan-Meier survival curves of BP903 tumour-beating animals, treated as in (A).

(D) Schematic representation depicting the treatment regimen.

(E) Tumour growth curves of BP903 T^{O}s: Control (n=7), diABZI/E (n=8), diABZI/αPD-1 (n=8), diABZI/A/E (n=10) and diABZI/A/E/αPD-1 (n=10). Each line represents one animal and thick lines represent average tumour growth.

(F) Tumour growth kinetics (days 0-28) of mice treated as in (D), measured by the area under the curve (AUC). Each point represents the AUC of individual mice from (E).

(G) Kaplan-Meier survival curves of BP903 T^{O} tumour-bearing animals, treated as in (D).

(H) Pie charts depicting the proportion of mice from (G), which progressed, partially responded, or fully responded to the indicated treatments.

(I) Tumour growth curves of BP487 T^{O}s: Control (n=9), diABZI/A/E (n=9) and diABZI/A/E/αPD-1 (n=9). Each line represents one animal and thick lines represent average tumour growth.

(J) Tumour growth kinetics (days 0-25) of mice treated as in (D), measured by the area under the curve (AUC). Each point represents the AUC of individual mice from (J).

(K) Kaplan-Meier survival curves of BP487 T^{O} tumour-bearing animals, treated as in (D).

(L) Pie charts depicting the proportion of mice from (K), which progressed, partially responded, or fully responded to the indicated treatments.

p values were calculated using Log-rank (Mantel-Cox) test (C, G, K) and ordinary one-way ANOVA with Tukey's multiple comparisons (F, J).

A=ASTX660; E=emricasan; diABZI=STING agonist

Source data are provided as a Source Data file and in

Zenodo: https://doi.org/10.5281/zenodo.18130193

Supplementary Fig. 9

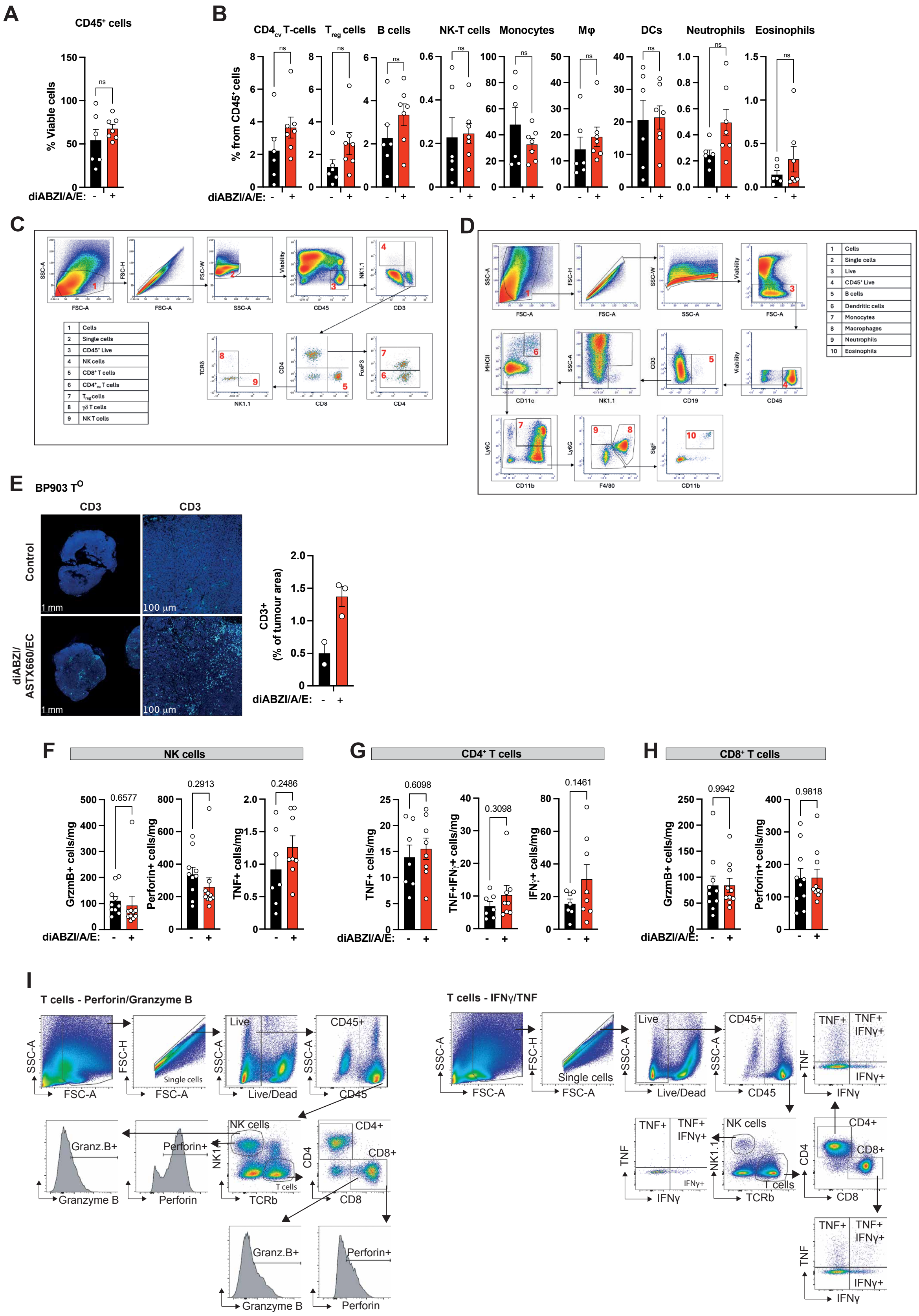

Supplementary Fig. 9. STING-driven necroptosis enhances tumour immunogenicity but fails to elicit durable adaptive immune response

(A) FACS analysis evaluating the presence of viable CD45+ cells in BP903 T^{O}s, following treatment with diABZI/ASTX660/E. Each dot represents an individual tumour.

(B) FACS analysis of tumours showing the percentage of $CD4_{conv}$ T-cells, T_{reg} cells, B-cells, NK-T cells, Monocytes, Mφ, DCs, Neutrophils and Eosinophils within the $CD45^{+}$ population, treated as in (Fig 6K), and harvested on day 12 post treatment. Control (n=6), diABZI/ASTX660/E (n=7). Each dot represents an individual tumour.

(C) FACS gating strategy, related to Fig 6L: 1) Cells, 2) Single cells, 3) $CD45^{+}$ live cells, 4) NK cells, 5) $CD8^{+}$ T-cells, 6) $CD4^{+}_{CV}$T-cells, 7) T_{reg} cells, 8) γδ T cells, 9) NK T cells.

(D) Flow cytometry gating strategy, related to Fig. 6N-Q: 1) Cells, 2) Single cells, 3) Live cells, 4) $CD45^{+}$ live cells, 5) B cells, 6) Dendritic cells, 7) Monocytes, 8) Macrophages, 9) Neutrophils, 10) Eosinophils.

(E) PhenoCycler analysis. Representative images from BP903 tumours treated as illustrated in Fig. 6K, showing T cell (CD3+) infiltration. BP903 T^{O}s were derived from two (Captisol) or three (diABZI/A/E) independent animals. Scale bar 1 mm and 100 µm. Data show mean ± SEM.

(F) FACS analysis of tumours showing GrzmB+, Perforin+ and TNF + NK cells. Each dot represents an individual tumour.

(G) FACS analysis of tumours showing TNF+,IFNγ and TNF+/IFNγ+ CD4+ T cells. Each dot represents an individual tumour.

(H) FACS analysis of tumours showing GrzmB+ and Perforin+ CD8+ T cells. Each dot represents an individual tumour.

(I) Flow cytometry gating strategy, related to F-H.

p values were calculated using unpaired t-test, two-tailed (A, B) and Welch's t test, two-tailed (F, G, H).

Source data are provided as a Source Data file and in

Zenodo: https://doi.org/10.5281/zenodo.18130193

Supplementary Fig. 10

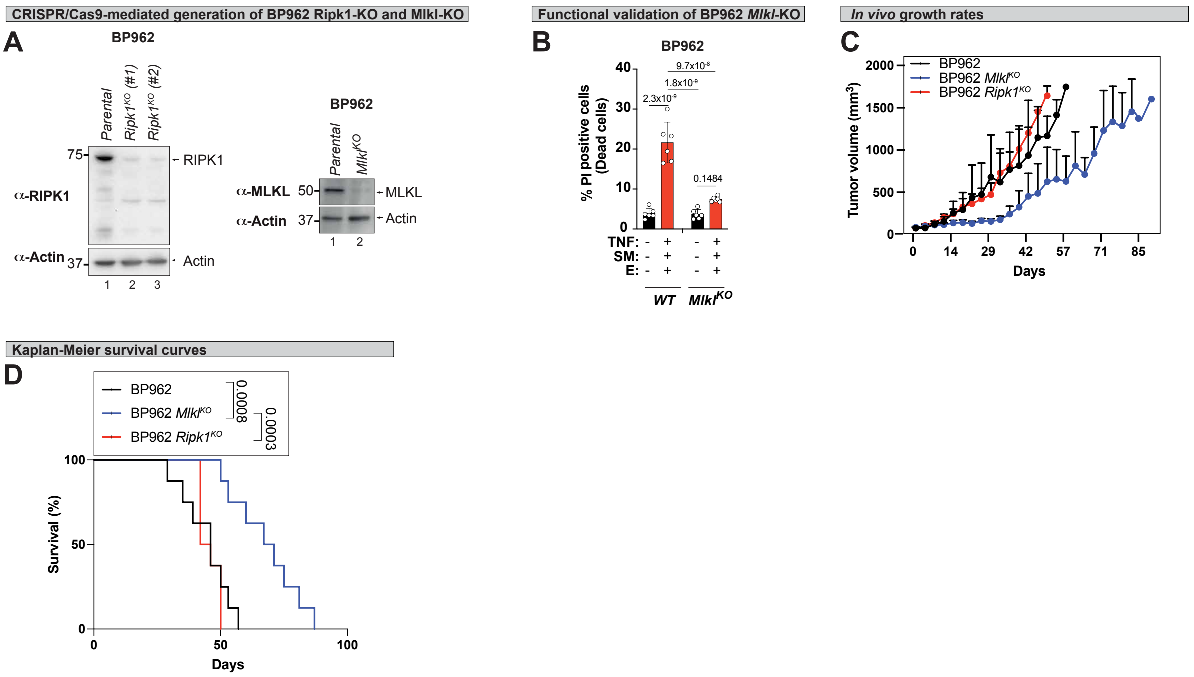

Supplementary Fig. 10. Generation and characterisation of *BP962*$^{\textit{Ripk1-KO}}$ and *BP962*$^{\textit{Mlkl-KO}}$ tumours

(**A**) Western blot validation of CRISPR-mediated RIPK1 and MLKL deletion in BP962 organoids. RIPK1 and MLKL knockout lines were generated and validated independently, each with its respective control. Samples derive from the same experiment. RIPK1 and Actin were run on the same gel, whereas MLKL and Actin were run on a separate gel processed in parallel. Images are representative of 2 independent biological repeats.

(B) Cell death assay confirming necroptosis deficiency in BP962$^{\textit{Mlkl}\text{-KO}}$ organoids. Each dot represents one independent biological replicate.

(C) Tumour growth comparison of the indicated organoid lines in C57BL/6J mice, 8 mice were injected for each organoid line.

(D) Kaplan-Meier survival curves of the indicated tumour-bearing animals from (C).

p values were calculated using Mann-Whitney (B) and Log-rank (Mantel-Cox) test (D).

Source data are provided as a Source Data file and in

Zenodo: https://doi.org/10.5281/zenodo.18130193
