# Supplementary material for: Necroptosis in both tumour and stromal compartments determines responsiveness to immunogenic cell death-based immunotherapy
Source: Nat Commun. 2026 Mar 6;17:3597. doi: 10.1038/s41467-026-70133-8 (PMC13096328; doi:10.1038/s41467-026-70133-8)
Supplement: Supplementary file 2 — Descriptions of Additional Supplementary Files [file 41467_2026_70133_MOESM2_ESM.pdf]

Descriptions of Additional Supplementary Files

Supplementary Data 1
This document provides an inventory of all key reagents and consumables used in the study, including (but not limited to) antibodies, chemicals and tissue-culture materials, oligonucleotides, Taqman probes, Opal 6-plex reagents, recombinant DNA, critical commercial assays, cell lines and organoids, organisms and strain information and software and algorithms. For each item, the document lists the relevant details required for traceability and reproducibility (e.g., supplier/manufacturer, catalogue number, dilutions).

Supplementary Data 2
This document provides the ID to source gene lists and GSEA pathway names used in the study. It also lists the genes included in the pathway.

Supplementary Data 3
This document provides the complete set gene names of IFN- and NF-κB pathway target genes used for the heatmap comparison shown in Fig. 3 J. For each gene, normalized expression values across the three different organoids are reported. Gene names are provided in full to facilitate interpretation of the heatmap, where labelling is too small because of size limit.
