# Supplementary material for: Necroptosis in both tumour and stromal compartments determines responsiveness to immunogenic cell death-based immunotherapy
Source: Nat Commun. 2026 Mar 6;17:3597. doi: 10.1038/s41467-026-70133-8 (PMC13096328; doi:10.1038/s41467-026-70133-8)
Supplement: Supplementary file 6 — Reporting Summary [file 41467_2026_70133_MOESM6_ESM.pdf]

nature portfolio

Corresponding author(s): Pascal Meier

Last updated by author(s): 27/01/2026

Reporting Summary

Nature Portfolio wishes to improve the reproducibility of the work that we publish. This form provides structure for consistency and transparency in reporting. For further information on Nature Portfolio policies, see our Editorial Policies and the Editorial Policy Checklist.

Statistics

For all statistical analyses, confirm that the following items are present in the figure legend, table legend, main text, or Methods section.

n/a	Confirmed	
☐	☒	The exact sample size (*n*) for each experimental group/condition, given as a discrete number and unit of measurement
☐	☒	A statement on whether measurements were taken from distinct samples or whether the same sample was measured repeatedly
☐	☒	The statistical test(s) used AND whether they are one- or two-sided<br>*Only common tests should be described solely by name; describe more complex techniques in the Methods section.*
☐	☒	A description of all covariates tested
☐	☒	A description of any assumptions or corrections, such as tests of normality and adjustment for multiple comparisons
☐	☒	A full description of the statistical parameters including central tendency (e.g. means) or other basic estimates (e.g. regression coefficient) AND variation (e.g. standard deviation) or associated estimates of uncertainty (e.g. confidence intervals)
☐	☒	For null hypothesis testing, the test statistic (e.g. *F*, *t*, *r*) with confidence intervals, effect sizes, degrees of freedom and *P* value noted<br>*Give P values as exact values whenever suitable.*
☒	☐	For Bayesian analysis, information on the choice of priors and Markov chain Monte Carlo settings
☒	☐	For hierarchical and complex designs, identification of the appropriate level for tests and full reporting of outcomes
☐	☒	Estimates of effect sizes (e.g. Cohen's *d*, Pearson's *r*), indicating how they were calculated

Our web collection on statistics for biologists contains articles on many of the points above.

Software and code

Policy information about availability of computer code

Data collection

Generation of organoid lines
Organoid lines were generated using an adaptation of a previously described method (Duarte et al. 2018). Organoids were derived from tumours that had spontaneously developed in Blg-Cre;Brca1f/fp53+/- mice.

Generation of CRISPR knockout Organoids
Guide RNA preparation was described before61. Organoids were broken down into single cells using TrypLe and 1x106 cells were seeded per well into Matrigel coated 6 well plates. After 24 h, cells were transiently transfected with Lipofectamine 3000 (Invitrogen). After 48 h, GFP positive cells were FACS sorted, seeded back into Matrigel and screened for gene knockout.

PCR genotyping
Organoids were homogenised using a Qiashredder (Qiagen). For ex vivo mouse tissues, samples were snap frozen immediately after dissection, then a 20-30 mg fragment of tissue was disrupted and homogenised using a Precellys bead mill (Bertin). DNA was extracted from the homogenised samplesusing the Dneasy Blood and Tissue Kit (Qiagen), as per manufacturer instructions. PCR reactions were
performed according to the mouse genotyping protocols published by The Jackson Laboratory (Jackson Laboratories 2019), using the KAPA2G Fast HotStart PCR Kit (Kapa Biosystems), with primers (see Suppl. Table 1). DNA electrophoresis was run in 2.5% agarose gels (Invitrogen) and gels were imaged using a ChemiDoc Touch Imaging System (Bio-Rad).

RT-qPCR
RT-qPCR was performed using two TaqMan assays (Thermo Fisher Scientific); one with the target sequence in exon 19 (present in all alleles) and one with the target sequence in exon 22 (which is present if the floxing event has not occurred, and absent if the floxing event has occurred)46. RT-qPCR was performed on RNA extracted from tail tips of wild-type C57BL/6J mice, organoids, and organoidderived tumours. Prior to RNA extraction, mouse tissues (tumours and tail tips) were mechanically disrupted and homogenised using a Precellys 24 tissue homogeniser (Bertin). For organoids 1x106 cells were collected and homogenised using a Qiashredder (Qiagen). RNA was extracted from homogenised samples using the RNeasy Mini Kit (Qiagen) as per manufacturer instructions. RNA quantity and quality were measured using a NanoDrop 8000 Spectrophotometer (Thermo Fisher Scientific). cDNA was synthesised from RNA using the High-Capacity cDNA Reverse Transcription Kit (Applied Biosystems). For Brca1 exon 19 and exon 22, qPCR was performed using TaqMan Gene Expression Assays (Applied Biosystems) as previously described46. RT-qPCR results were analysed using the DDCt method. Ct values were calculated from triplicate repeats for each sample. The Ct value of a housekeeping gene (bActin) was subtracted from the Ct value of each sample, to calculate the DCt value. The DCt value for each sample was then normalised to a control sample by subtraction to calculate DDCt. The fold change in expression was calculated as the negative exponent of this value (2-DDCt). RNA was extracted from homogenised BP903 tumour fragments using the AllprepÒ DNA/RNA Micro kit (Qiagen) and converted to cDNA using QuantiTec Reverse Transcription Kit (Qiagen), according to manufacturer's instructions. RT-qPCR and data analysis were performed as previous described112. For BP903 TOs, mRNA was extracted 12 days post treatment with a single injection of vehicle control or diABZI/ASTX660/E. The relative mRNA expression of indicated genes was measured using Taqman probes (Thermo Fisher Scientific): Actb (House-keeping gene (Mm00607939_s1), Ifng (Mm01168134_m1), Perforin (Mm00812512_m1), and Ccl5 (Mm01302427_m1).

Bulk RNA-seq
RNA was extracted using the AllprepÒ DNA/RNA Micro kit (Qiagen) from snap frozen samples of the primary tumour, early passage established normal and tumour organoids (passage number 10-20), organoid-derived tumours, as well as normal murine mammary fat pads. Three samples were analysed per group. For organoids, 1x106 cells were collected and homogenised using a Qiagen Qiashredder. For mouse tissue, samples were snap frozen immediately after dissection, then a 20-30 mg fragment of tissue was disrupted and homogenised using a Precellys bead mill (Bertin). RNA quantity and quality were determined using the Nanodrop spectrophotometer (Thermo Fisher Scientific). Strand-specific mRNA libraries were prepared using the NEBNext Ultra II Directional RNA Library Prep Kit for Illumina (NEBNext Poly(A) mRNA Magnetic Isolation Module; New England Biolabs), from 500 ng of RNA per sample. The quantity and quality of the libraries were analysed using the 2100 Bioanalyzer system (Agilent). Sequencing was performed using the Illumina NovaSeq 6000, on an S1 100 bp PE Nova flowcell, to achieve 7.5 Gigabases per sample.

Opal 6-plex
3 µm FFPE tissue sections were baked for 1 h and run on the Leica Bond Rx platform. Several cycles of sequential staining were conducted by using Epitope Retrieval solution 1 for 20 minutes between each antibody, apart for the last antibody in the sequence where Epitope Retrieval solution 2 was used for 30 minutes. Antibodies were applied with Opalä pairings in the following order: Panel 1 – Lyve1 (ab33682) 1:1500 with Opal 520 1:300, F4/80 (ab300421) 1:1000 with Opal 690 1:150, CD31 (ab182981) 1:1000 with Opal 480 1:100, Ki67 (ab15580) 1:1000 with Opal 570 1:300, CD45 (70257S) 1:100 with Opal 620 1:300 and CD19 (ab245235) 1:200 with TSA-DIG 1:100 followed by anti-DIG-Opal780 1:25. Panel 2 – Ly6G (87048S) 1:100 with Opal 520 1:300, F4/80 (ab300421) 1:1000 with Opal 690 1:150, aSMA (M0851) 1:250 with Opal 570 1:300, CD3 (ab134096) 1:500 with Opal 480 1:100, CD45 (70257S) 1:100 with Opal 620 1:300 and CD19 (ab245235) 1:200 with TSA-DIG 1:100 followed by anti-DIG-Opal780 1:25. Bond anti-rabbit Polymer was used as the secondary antibody for antibodies raised in rabbit. Horse anti-Mouse IgG (Rat adsorbed)-Biotinylated secondary antibody (BA-2001) 1:400 and Streptavidin-Peroxidase (P0397) 1:500 were used as the secondary antibodies for antibodies raised in mouse. Slides were scanned using the PhenoImager HT (formerly Vectra Polaris). Spectral unmixing and auto-fluorescence removal were performed using the Phenochartä and Informä softwares.

Histopathology, sample preparation and staining
Tumour material was fixed in 10% neutral buffered formalin for 24 h. Subsequently, tumours were washed in PBS followed by processing from 50% EtOH through to embedding in paraffin wax. Organoids were fixed in 10% neutral buffered formalin for 15 minutes, embedded in Histogel (Richard-Allan Scientific) then processed to wax embedding as above. For IHC staining, 4 µm sections were cut onto adhesive slides, air dried and baked at 60oC to enhance adhesion and stored at 4oC prior to staining. Prior to IHC staining, heat induced epitope retrieval (HIER) and deparaffinisation was performed using the Dako PT LINK module for 20 minutes at 97oC with a 20 minute warmup and then 20 minute cooldown to 65oC, in either pH 6 or pH 9 Dako Target Retrieval Solution (Agilent Technologies). Endogenous peroxidases were blocked using Agilent REAL peroxide block. All staining was performed on the Dako Link48 or Ventana Discovery Ultra automated immunostaining platform, which, for RIPK1, was similar to the one described in127. For staining carried out on the Link48 platform: For primary antibodies raised in mouse, endogenous mouse immunoglobulin was blocked using M.O.M. (Mouse on Mouse) Blocking Reagent (Vector Laboratories) and non-specific interactions blocked using Dako Protein Block (Agilent) prior to application of the primary antibody. Rat primary antibodies were detected using Rat Histofine Reagent (Nichirei Biosciences), rabbit primary antibodies using ImmPRESS HRP Horse anti-Rabbit IgG (Vector Laboratories), and the mouse primary antibody using

EnVision anti-mouse-HRP reagent (Agilent). For staining carried out on the Ventana Discovery slides were deparaffinised using Roche Discovery Wash and HIER was performed for 64 minutes at 95OC using CC1 (high pH) buffer. Endogenous peroxidase was blocked using ChromoMap Inhibitor CM (Roche). Primary antibodies were diluted in Ventana Primary Antibody Diluent with casein protein block (Roche) and applied manually. Slides were incubated at room temperature for 32 minutes before incubation with UltraMap anti-Rabbit HRP secondary multimer for 8 minutes. Slides were visualised using Roche ChromoMap and haematoxylin counterstain. Slides were digitised using a Nanozoomer XR (Hamamatsu) using a x20 dry objective and the ndpi images imported into QuPath128 for digital analysis.

Data analysis

Copy-number analysis
Copy-number aberrations were calculated using CNVkit (v0.9.9113) and Control-FREEC (v11.5114). For CNVKit, gains/amplifications and losses/deletions were defined as log2ratio > 0.5 and log2ratio < -0.3, respectively. For Control-FREEC, default gain/amplification and loss/deletion filters were used. Results from the two callers were converted to R GRanges objects and subsequently intersected keeping the common copy-number calls only. Preprocessing of whole exome sequencing dataset was performed as previously described using the mouse reference genome (GRCm38).

Computational analysis of RNA sequencing data
For raw sequence quality control, FastQC and FastQ Screen were run on all sample FASTQ files, and a summarised report was generated using MultiQC116,117. FASTQ reads were trimmed using Trim Galore (v0.6.6). Paired-end reads (100 bp long) were aligned to the mouse reference genome (version GRCm38), using STAR (v2.7.6a) with quantMode GeneCounts and twopassMode Basic alignment settings118. The annotation file used for feature quantifications was downloaded from GENCODE (v17)in GTF file format. Differential mRNA abundance analysis was performed using the edgeR package119
in R (version 3.6.0). Results were annotated using ENSEMBL gene annotations from R package org.Mm.eg.db. Genes were considered statistically significant if the log2 fold change was greater than 1, and the false discovery rate-adjusted p value was below 0.05. Principal component analysis (PCA) was performed using custom libraries in R statistical environment (v.3.6.0). Differences in the expression of stromal elements was interrogated using the stroma-derived prognostic predictor (SDPP) gene set120. Samples were classified into breast cancer subtypes based on their gene expression profiles using the AIMS gene classifier121. A mouse-specific mammary tumour classifier was created using centroids of a previously published microarray dataset122. The MHC class I antigen presentation pathway was analysed by comparing the differential expression of various organoids, using a gene list from Nanostring's pan-cancer/immune gene sets (https://nanostring.com/products/ncounter-assayspanels/oncology/pancancer-immune-profiling/). The immune checkpoint gene expression was analysed by comparing the differential expression of the indicated organoids.

Gene set enrichment analyses (GSEA)
Macrophage-related gene sets were obtained from MSigDB123 by querying mouse collections for the keyword "macrophage" and further filtering to include only those containing the term in their name. Gene Set Enrichment Analysis (GSEA) was performed in R 4.2.1 with ClusterProfiler using fold-change values from differential expression comparisons of different TOs.
Cell type deconvolution with CIBERSORTx
To perform cell type deconvolution of bulk RNA samples, cell-type specific transcriptomes were obtained from the Brca1-/-p53-/- TNBC mouse model124. scRNA-seq counts were converted to transcripts per million (tpm) and the dataset was down sampled to 2500 cells to increase computing efficiency. A signature matrix was then created using a CIBERSORTx125 singularity container with default settings. Subsequently, fractions mode was employed for cell type proportion deconvolution using relative-mode, 100 per mutations, rmbatchSmode and disabling quantile normalisation.

Cell death assay with organoids in vitro using ImageXpress
To image cell death (PI uptake) of
organoids, we used the ImageXpress Confocal High-Content Imaging System (Molecular Devices) with a Nikon Plan Fluor Ph1 DLL 10x/ 0.3 NA air objective. The system is equipped with a Lumencor LED light source and an Oxford Nanoinstruments Zyla camera. Images of the Hoesht (405 nm ex/461 nm em) and PI (560 nm ex/ 615 nm em) were captured at 400 ms and 300 ms exposures, respectively, with 2 or 4 fields of view imaged per well and a z-stack of 5 µm steps over ~150 µm range, which was subsequently compressed to 2D maximum intensity projections. A custom module workflow was built using the MetaXpress analysis software (Molecular Devices). The workflow consisted of 9 steps. Step 1: ‘Setup’ module defined the channels used for analysis, ‘DAPI’ and ‘Texas Red’. Step 2: ‘Simple Threshold’ module to segment the organoid structures using the DAPI channel to create an object mask of the organoid total area. The threshold was adjusted as required depending on signal intensity. Step 3: ‘Filter Mask’ module determined the size of organoid structures (or objects) to be included foranalysis, and enabled exclusion of debris or single cells and adjusted as required between experiments.
Step 4: ‘Grow Objects’ module followed by Step 5: ‘Fill Holes' module improved segmentation by merging nuclei and filling in any space created by the presence of a lumen, to create complete objects for the mask. For the Grow objects, we consistently increased by 3 pixels. Step 6: ‘Remove Round Objects’ module removed all partially segmented organoids obstructed by the edge of the imaging field of view to complete the first organoid mask. Step 7: ‘Find Round Objects’ module was used to segment PI positive nuclei using thresholding on channel 2 to create the second mask of organoid cell death.
Step 8: ‘Grow Objects’ module expanded the segmented PI positive objects by 3 pixels. Step 9: ‘Measure Mask’ module measured the total area of PI stain overlayed within the defined segmented

total viable objects from channel 1. The above analysis pipeline outputs a large table containing the total area measurement for both the first organoid mask (total organoid area) and second organoid cell death mask (PI area only) for every object measured, as well as other related data. To automate and streamline the analysis, a python script was written in Jupyter Notebook to clean up the data table, calculate the mean PI/DAPI ratio per field of view and export the rearranged table into a format suitable for downstream analysis.
Histopathology analysis
Morphological assessment of essential pathological features of primary and organoid-derived tumours was made on digital scans of H&E sections. Features defining tumour grade (tubule formation, nuclear pleomorphism, mitotic count) were evaluated along guidelines for clinical purposes, as defined by the Royal College of Pathologists (RCPath). Additional characteristics of the tumour and tumour microenvironment, including tumour necrosis, stromal features and immune infiltrates, were alsoassessed. A semi-quantitative assessment of RIPK1 expression on primary tumours and normal lobules
was carried out by applying the H-score method, which incorporates both staining intensity and percentage of stained cells at each intensity level as follows: H-score = (0 x % negative cells) + (1 x % weak positive cells) + (2 x % moderate positive cells) + (3 x % strong positive cells), with overall score ranging from 0 to 300. For semi-quantitative assessment of ER and PR expression, the Allred score (0-8 Quick score) was employed, in keeping with the recommended by the RCPath method for clinical practice (score for proportion: 0 = no staining, 1 = < 1% nuclei staining, 2 = 1–10% nuclei staining, 3 = 11–33% nuclei staining, 4 = 34–66% nuclei staining, 5 = 67–100% nuclei staining; score for intensity: 0 = no staining, 1 = weak staining, 2 = moderate staining, 3 = strong staining). Current consensus is that the recommended cut-off point for positivity versus negativity for ER status is ≥1% of tumour cells. For HER2 expression, the semi-quantitative method, which is based on the intensity of staining and percentage of membrane positive cells, recommended by RCPath was employed. This method gives a score range of 0 to 3+, with samples scoring 3+ regarded as positive, and those scoring 0/1+ as negative. Borderline scores (2+) are regarded as equivocal. For the QuPath quantification of stroma to tumour ratio on primary and organoid-derived tumours, H&Estained images were manually annotated with areas of "Stroma" and "Tumour." Whitespace was classified as "*Ignore". These annotations were used to train a pixel classifier using Random Trees at a resolution of 1.81 µm/pixel and utilising all multiscale features. The area occupied by each was used to calculate the stroma to tumour ratio in each specimen. The luminal space was calculated by subtracting the area classified as "Tumour" and the area classified as "Stroma" from the parent annotation area. For the QuPath quantification of infiltration of organoid-derived tumours by F4/80 positive cells, IHC images had their haematoxylin and DAB colour vectors automatically calculated, and the DAB channel was threshold at an Optical Density of 0.1 to create a binary mask. The tumours were manually annotated to include only tumour mass and exclude surrounding tissues. The total area of tumour occupied by positive pixels was calculated. For the QuPath quantification of infiltration of organoid-derived tumours by CD163 or CD8 positive cells, IHC images had their haematoxylin and DAB colour vectors automatically calculated and the stains deconvoluted. The tumour boundary was annotated manually using the polygon annotation tool and surrounding tissues and necrotic regions were excluded. The number of positive cells within the tumour mass (intratumoral area) was quantified using Positive cell detection plug-in. The sum optical density (OD) channel was used for cell detection at a threshold of 0.1. Cells were classified as Positive when the mean DAB OD exceeded 0.07. To count positive cells surrounding the tumour boundary (peritumoral area), the tumour boundary annotation was expanded by 100 µm and 50 µM for CD8 and CD163, respectively. For the QuPath quantification of PD-L1 positive cells, the DAB mean is the average of DAB stain optical density values (at a 2 µm resolution per pixel) across the annotation area (not multiple fields of view). The annotation area for these scans was the area covered by tumour cells, leaving out the surrounding tissue (adipose/lymph node etc).

For manuscripts utilizing custom algorithms or software that are central to the research but not yet described in published literature, software must be made available to editors and reviewers. We strongly encourage code deposition in a community repository (e.g. GitHub). See the Nature Portfolio guidelines for submitting code & software for further information.

Data

Policy information about availability of data

All manuscripts must include a data availability statement. This statement should provide the following information, where applicable:

- Accession codes, unique identifiers, or web links for publicly available datasets
- A description of any restrictions on data availability
- For clinical datasets or third party data, please ensure that the statement adheres to our policy

All sequencing data set have been deposited to SRA under submission ID=SUB15008798. This dataset will be released under acceptance.

Research involving human participants, their data, or biological material

Policy information about studies with human participants or human data. See also policy information about sex, gender (identity/presentation), and sexual orientation and race, ethnicity and racism.

Reporting on sex and gender	N/A
Reporting on race, ethnicity, or other socially relevant groupings	N/A

Population characteristics	N/A
Recruitment	N/A
Ethics oversight	N/A

Note that full information on the approval of the study protocol must also be provided in the manuscript.

Field-specific reporting

Please select the one below that is the best fit for your research. If you are not sure, read the appropriate sections before making your selection.

☒ Life sciences ☐ Behavioural & social sciences ☐ Ecological, evolutionary & environmental sciences

For a reference copy of the document with all sections, see nature.com/documents/nr-reporting-summary-flat.pdf

Life sciences study design

All studies must disclose on these points even when the disclosure is negative.

Sample size	All experiments were performed with minimum of 3 biological replicates as acceptable in this field
Data exclusions	None
Replication	All experiments were performed with minimum of 3 biological replicates as acceptable in this field
Randomization	N/A
Blinding	N/A

Reporting for specific materials, systems and methods

We require information from authors about some types of materials, experimental systems and methods used in many studies. Here, indicate whether each material, system or method listed is relevant to your study. If you are not sure if a list item applies to your research, read the appropriate section before selecting a response.

Materials & experimental systems

n/a	Involved in the study	
☐	☒	Antibodies
☐	☒	Eukaryotic cell lines
☒	☐	Palaeontology and archaeology
☐	☒	Animals and other organisms
☒	☐	Clinical data
☒	☐	Dual use research of concern
☒	☐	Plants

Methods

n/a	Involved in the study	
☒	☐	ChIP-seq
☐	☒	Flow cytometry
☒	☐	MRI-based neuroimaging

Antibodies

Antibodies used

CD19 BD Biosciences 563148 BV605 1D3
CD45 BD Biosciences 563410 BV650 30-F11
CD64 Biolegend 139311 BV711 X54
CD4 BD Biosciences 740007 BV421 RM4-5
Ly6G BD Biosciences 560602 PerCP-Cy5.5 1A8
SigF BD Biosciences 566211 BB515 E50-2440
Ly6C BD Biosciences 562728 PE-CF594 AL-21
CD11c BD Biosciences 561022 Pe-Cy7 HL3
F4/80 BD Biosciences 565410 PE T45-2342
MHCII Biolegend 107621 AF700 M5/114.15.2
NK1.1 Biolegend 108723 APC-Cy7 PK136
Tim4 eBiosciences 46-5866-80 PerCP-eFluor710 RMT4-54
CD8 BD Biosciences 612759 BUV737 53-6.7
CD11b BD Biosciences 565976 BUV395 M1/70
CD19 BD Biosciences 563148 BV605 1D3
CD45 BD Biosciences 563410 BV650 30-F11
CD64 Biolegend 139311 BV711 X54-5/7.1

CD4 BD Biosciences 740007 BV421 RM4-5
Ly6G BD Biosciences 740157 BV510 1A8
SigF BD Biosciences 566211 BB515 E50-2440
Ly6C BD Biosciences 562728 PE-CF594 AL-21
CD11c BD Biosciences 561022 Pe-Cy7 HL3
F4/80 eBioscience 12-4801-80 PE BM8
MHCII eBioscience 56-5321-82 AF700 M5/114.15.2
NK1.1 BD Biosciences 741477 BUV661 PK136
TCRg/d BD Biosciences 748993 BUV563 GL3
CD3 BD Biosciences 561826 APC 145-2C11
CD8 BD Biosciences 612759 BUV737 53-6.7
CD11b BD Biosciences 565976 BUV395 M1/70
PD-1 BD Biosciences 563059 BV605 J43
CD45 BD Biosciences 563410 BV650 30-F11
CD62L BD Biosciences 740660 BV711 MEL-14
CD4 BD Biosciences 740007 BV421 RM4-5
Ki-67 Biolegend 417-5698-82 BV786 SolA15
CD25 Biolegend 102005 FITC PC61
CD44 BD Biosciences 562464 PE-CF594 IM7
CD69 BD Biosciences 561930 Pe-Cy7 H1.2F3
Foxp3 eBioscience 12-5773-80 PE FJK-16s
CD3 Biolegend 100215 AF700 17A2
NK1.1 BD Biosciences 741477 BUV661 PK136
TCRg/d BD Biosciences 748993 BUV563 GL3
GranzymeB Biolegend 515405 AF647 GB11
Total MLKL CST 26539
Phospho-MLKL CST 37333
Phospho-STAT1 CST 9167
Total STAT1 CST 9172
Phospho-IRF3 CST 4947
Total IRF3 CST 4302
GAPDH CST 2118
RIPK1 CST 3493
RIPK3 CST CST
Phalloidin-633 Thermo Fischer Scientific A22284
ECAD BD Biosciences 610182
Cytokeratin 14 Biolegend 905301 polyclonal pH6 (1:4000) Rabbit ImmPRESS (Vector) Dako Link48 Autostainer
ER Leica NCL-L-ER-6F11 6F11 pH9 (1:40) Mouse EnVision (Agilent) Dako Link48 Autostainer
PR Cell signalling 8757 D8Q2J pH9 (1:500) Rabbit ImmPRESS (Vector) Dako Link48 Autostainer
HER2 CST 2165 29D8 pH9 (1:5000) UltraMap anti-Rabbit HRP Ventana Discovery Ultra
F4/80 Bio-Rad MCA497G A3-1 pH6 (1:100) Rat N-Histofine (Nichirei) Dako Link48 Autostainer
Lyve1 Abcam ab33682
F4/80 Abcam ab300421
CD31 Abcam ab182981
Ki67 Abcam ab15580
CD45 Cell Signaling Technology 70257S
CD19 Abcam ab245235
Ki67 CellMarque 275R SP6 pH6 (1:100) Rabbit ImmPRESS (Vector) Dako Link48 Autostainer
CD8a AbCam ab217344 EPR21769 pH9 (1:2000) Rabbit EnVision (Agilent) Dako Link48 Autostainer
CD163 AbCam ab182422 EPR19518 pH9 (1:500) UltraMap anti-Rabbit HRP Ventana Discovery Ultra
PD-L1/B7-H1 R&D systems (Bio-techne) AF1019-SP polyclonal pH9 (1:25) UltraMap anti-Rabbit HRP Ventana Discovery Ultra
ECAD Agilent Dako M3612 NCH-38 pH9 (1:50) Mouse EnVision (Agilent) Dako Link48 Autostainer
RIPK1 CST 3493 D94C12 pH9 (1:50) Rabbit EnVision (Agilent) Dako Link48 Autostainer
Ly6G Cell Signaling Technology 87048S
F4/80 Abcam ab300421
aSMA Dako (Agilent) M0851
CD3 Abcam ab134096
CD45 Cell Signaling Technology 70257S
CD19 Abcam ab245235
CD8b.2 (BD Biosciences, BUV805, clone 53-5.8)
TCRβ (BioLegend, PerCP-Cy5.5, clone H57-597)
Perforin (BioLegend, PE, clone S16009A)
IFNγ (BioLegend, AF647, clone XMG1.2)
TNF (BioLegend, PE, clone MP6-XT22)

Validation

All aforementioned antibodies have been already validated and have been previously used in other publications. This information can be found in their respective webpage.

Eukaryotic cell lines

Policy information about cell lines and Sex and Gender in Research

Cell line source(s)

EO771 CH3 Biosystems #94A001
BP110 This Manuscript N/A
BP111 This Manuscript N/A

	BP118 This Manuscript N/A<br>BP487 This Manuscript N/A<br>BP648 This Manuscript N/A<br>BP649 This Manuscript N/A<br>BP774 This Manuscript N/A<br>BP901 This Manuscript N/A<br>BP902 This Manuscript N/A<br>BP903 This Manuscript N/A<br>BP906 This Manuscript N/A<br>BP962 This Manuscript N/A<br>BP903MLKL-/- This Manuscript N/A<br>BP962RIPK1-/- This Manuscript N/A<br>BP962MLKL-/- This Manuscript N/A
Authentication	Cell lines were authenticated using STR profiling with the Geneprint10 Kit (Promega).
Mycoplasma contamination	Cell lines were tested monthly for mycoplasma infection and results were negative (<0.9).
Commonly misidentified lines<br>(See ICLAC register)	N/A

Animals and other research organisms

Policy information about studies involving animals; ARRIVE guidelines recommended for reporting animal research, and Sex and Gender in Research

Laboratory animals	All animal procedures were conducted within the guidelines of UK Home Office in accordance with Animals (Scientific Procedure) Act (ASPA) 1986, amended 2012 and the institutional guidelines of the Institute of Cancer Research. The Animal Welfare Ethical Review Body (AWERB) reviewed the protocols within the project license. All the animal experiments were conducted in accordance with the Animal Research: Reporting of In vivo Experiments (ARRIVE) guidelines to ensure reproducibility and transparency129. Blg-Cre;Brca1f/flp53-+- (C57BL/6) mice were aged until 12 to 15 months, with some mice undergoing at least one round of pregnancy and assessed for spontaneous tumour development in the mammary glands. Mice were culled when the tumours reached an average size of 12 to 15 mm and were processed to generate organoids as described above. Female C57BL/6J, MLKL-/- and NSG (NOD.Cg-Prkdcscid Il2rgtm1Wjl/SzJ) mice, aged between 6 to 8 weeks were used for tumour studies, including therapeutic interventions (only C57BL/6J). Mice were purchased from Charles River and enrolled in the study post an acclimatization period of at least one week.
Wild animals	This study did not involve wild animals
Reporting on sex	The study was done using mouse Breast cancer model using Blg-CRE driver that is expressed in luminal epithelial cells in mammary gland and as such was done with female mice
Field-collected samples	This study did not involve field collected samples
Ethics oversight	N/A

Note that full information on the approval of the study protocol must also be provided in the manuscript.

Plants

Seed stocks	N/A
Novel plant genotypes	N/A
Authentication	N/A

Flow Cytometry

Plots

Confirm that:

☒ The axis labels state the marker and fluorochrome used (e.g. CD4-FITC).

☒ The axis scales are clearly visible. Include numbers along axes only for bottom left plot of group (a 'group' is an analysis of identical markers).

☒ All plots are contour plots with outliers or pseudocolor plots.

☒ A numerical value for number of cells or percentage (with statistics) is provided.

Methodology

Sample preparation	Flow cytometry analysis<br>To determine immune infiltration, tumours were harvested and single cell suspensions were prepared. To this end, tumours were perfused with 1 mL/200 mg digest buffer (1 mg/ml Collagenase II (Merck), 0.1 mg/ml DNAse I (Merck), and 0.1% BSA in RPMI) before being minced into 1-2 mm pieces and incubated at 37°C for 1h shaking at 180 rpm. Digestion was inactivated with 20 mL MACS buffer (Miltenyi autoMACS running buffer Miltenyi, 130-091-221), and tissue suspension aggravated through a 70 µm filter. Cell suspensions were spun down at 350 rpm for 15 minutes at 4°C, and supernatant aspirated. Cell pellets were resuspended in 500 ml red blood cell lysis buffer (1x BD PharmLyse 555899 in H2O) and incubated at RT for 2 minutes. Lysis was inactivated with 2 mL HBSS and cells pelleted at 350 rpm for 6 minutes at 4°C, before aspiration of supernatant. Pelleted cells were resuspended in 500 µl viability dye (1:2000 dilution eBioscience™ Fixable Viability Dye eFluor™ 506, Thermofisher 65-0866-14 in HBSS) and incubated in the dark at RT for 20 minutes. Cells were washed with 2 mL MACS buffer and pelleted at 350 rpm for 6 minutes at 4°C, before aspiration of supernatant. Pelleted cells were resuspended in 50 µl Fc block (1:50 dilution in MACS buffer of Purified Rat Anti-mouse CD16/CD32, BD Biosciences 553142), and incubated in the dark at 4°C for 20 minutes. Antibody cocktail was prepared in a staining volume of 50 µl MACS buffer and added directly to cells in Fc block, prior to a further 30 minutes incubation. Stained cells were washed with 300 µl MACS buffer and pelleted as before. Cell pellets were fixed in 2% PFA in MACS buffer, in the dark at 4°C for 20 minutes. Finally, cells were washed as before, and resuspended in MACS buffer for flow cytometry analysis. 123 count eBeads (Thermofisher 01-1234-42) were added to each sample to calculate cell counts. To quantify changes in the immune landscape following treatment, tumours were harvested in ice-cold PBS from mice 12 days after treatment. Tumours were mechanically dissociated with scissors and enzymatically digested (30 minutes, 37°C), in PBS containing Trypsin-Versene (in house), 0.5 mg/ml Collagenase type I (Sigma, cat# C2674), 400 µg/ml Dispase type II (Sigma, cat# D4693), 1 mg/ml DNase type I (Roche, cat# 10104159001). Tumour suspensions were passed through a cell strainer (70 µm) into PBS (2% FBS & 2 mM EDTA), before centrifugation (1500 rpm, 10 minutes, 4°C). Pellets were resuspended in PBS-FBS 2% + Fc block (1:100, CD16/CD32, BD Biosciences cat# 553142) for 10 minutes (4°C), before surface staining for 30 minutes at 4°C with the appropriate antibody cocktail containing the viability dye (1:1000 eBioscience™ Fixable Viability Dye eFluor™ 780, cat# 65-0865-18). Cells were either fixed in 2% PFA for 20 minutes or fixed and permeabilised following the manufactured protocol (eBioscience, cat# 00-5523-00) prior staining with anti-Granzyme B, anti-Foxp3 and Ki67 antibodies.
Instrument	Flow cytometric analyses were carried out with a FACSymphony A5 (BD Biosciences) with FACSDiva software. 123 count eBeads (ThermoFisher, cat# 01-1234-42) were added to each sample to calculate cell counts.
Software	Data were analysed with FCS Express 7 software
Cell population abundance	N/A
Gating strategy	Shown on the figure

☒ Tick this box to confirm that a figure exemplifying the gating strategy is provided in the Supplementary Information.
